# MapTurns: mapping the structure, H-bonding, and contexts of beta turns in proteins

**DOI:** 10.1093/bioinformatics/btae741

**Published:** 2024-12-16

**Authors:** Nicholas E Newell

**Affiliations:** Independent Scientist, Reading, MA 01867, United States

## Abstract

**Motivation:**

Beta turns are the most common type of secondary structure in proteins after alpha helices and beta sheets and play many key structural and functional roles. Turn backbone (BB) geometry has been classified at multiple levels of precision, but the current picture of side chain (SC) structure and interaction in turns is incomplete, because the distribution of SC conformations associated with each sequence motif has commonly been represented only by a static image of a single, typical structure for each turn BB geometry, and only motifs which specify a single amino acid (e.g. aspartic acid at turn position 1) have been systematically investigated. Furthermore, no general evaluation has been made of the SC interactions between turns and their BB neighborhoods. Finally, the visualization and comparison of the wide range of turn conformations has been hampered by the almost exclusive characterization of turn structure in BB dihedral-angle (Ramachandran) space.

**Results:**

This work introduces MapTurns, a web server for motif maps, which employ a turn-local Euclidean-space coordinate system and a global turn alignment to comprehensively map the distributions of BB and SC structure and H-bonding associated with sequence motifs in beta turns and their local BB contexts. Maps characterize many new SC motifs, provide detailed rationalizations of sequence preferences, and support mutational analysis and the general study of SC interactions, and they should prove useful in applications such as protein design.

**Availability and implementation:**

MapTurns is available at www.betaturn.com. Sample code is available at: https://github.com/nenewell/MapTurns/tree/main.

## 1 Introduction

Beta turns (Venkatachalam 1968), in which the protein backbone (BB) abruptly changes direction over four amino acid (AA) residues, represent the most common type of protein secondary structure after alpha helices and beta sheets and play key structural and functional roles (for an overview, see *Roles of beta turns in proteins* at www.betaturn.com). Previous work, reviewed briefly in D’Arminio *et al.* (2024) and Shapovalov *et al.* (2019), has developed beta-turn classification systems at multiple levels of precision in the Ramachandran space of a turn’s BB dihedral angles, partitioning the turns into types defined by angular ranges (Hutchinson and Thornton 1996) or derived by BB clustering (Shapovalov *et al.* 2019), and sequence preferences in turns have been computed for these categories. Some of these preferences have been rationalized with reference to the intrinsic properties of the side chains (SCs) involved (e.g. hydrophilicity, common in turns because they often lie close to the solvent-accessible surface) or intra-turn interactions (commonly hydrogen bonds) in which the SCs take part, but the distribution of SC structures associated with each sequence motif has generally been represented at most by a single, “typical” conformation for each BB geometry, illustrated with a static image. Furthermore, there has been no systematic analysis of SC motifs in beta turns that involve more than one AA, or motifs that link turns to their local BB neighborhoods, likely stabilizing the turns and the structural motifs in which they are found. The current picture of SC structure in beta turns and their local contexts is therefore only a rough sketch.

## 2 Tool description

This work introduces MapTurns, a server for motif maps, which are 3D graphical, interactive conformational heatmaps of the BB and SC structure and H-bonding associated with individual sequence motifs in four-residue turns and their two-residue N-/C-terminal BB neighborhoods (their “tails,” which represent turn contexts). A sequence motif is defined here simply as the occurrence of one or more particular AAs at specific positions in the turn or its tails, regardless of whether the motif is associated with a recurrent SC structure, which is termed a “SC motif” (single-AAs are included in the motif definition because many of the most important SC motifs in turns involve individual AAs). Maps are produced not only for beta turns, but also for four-residue “strand” turns that are not included in the beta turn definition (Shapovalov *et al.* 2019) because one or both of their central residues lie within beta strands.

Maps for beta turns are generated from a redundancy-screened dataset of 102,192 turns/tails (see Supplementary Section S1.1) by a three-stage, hierarchical clustering procedure (Supplementary Section S1.3), which uses a previously derived Ramachandran-space BB clustering generated by a hybrid DBSCAN/k-medoids algorithm (Shapovalov *et al.* 2019) as its first stage. In the second clustering stage, the turns within each BB cluster which contain the sequence motif are clustered by the conformations of the motif's SC(s), using a *k*-medoids PAM (partitioning around medoids) algorithm (Kaufman and Rousseeuw 2005) in Euclidean space. Finally, in the third clustering stage, the N- and C-terminal tails of the turns within each SC cluster are independently clustered in Euclidean space using *k*-medoids PAM.

In the maps for strand turns, which are classified into three groups {E2, E3, E2E3} depending on which of a turn’s two central residues lie within the strand, BB clusters are derived from a new *k*-medoids PAM clustering in Ramachandran space, since strand turns were not included in the beta-turn BB clustering (Shapovalov *et al.* 2019). SC and tail clusters for strand turns are generated using the same methods applied for beta turns.

**Figure 1.**
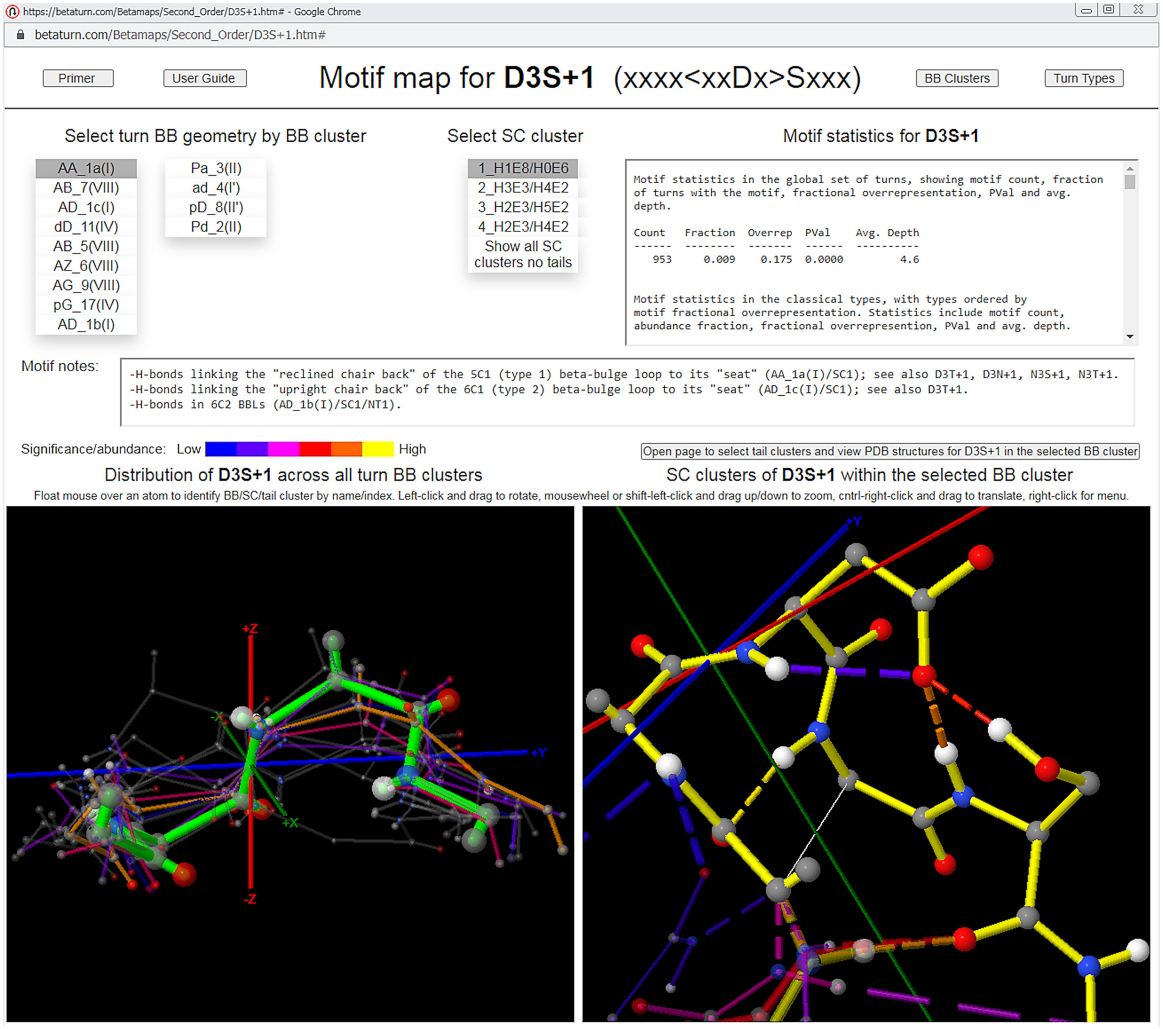
Upper-level motif map screen for the sequence motif that specifies Asp at turn position 3 and Ser just after the turn (D3S + 1). The upper-level motif map screen supports the exploration of the recurrent BB and SC structures and H-bonds associated with a sequence motif in beta turns. BB and SC clusters are represented by their medoids, which are color-coded with a heatmap of relative motif statistical significance (for BB clusters, at left, with the selected cluster highlighted) or cluster size (for the SC clusters within the selected BB cluster, at right) and scaled according to cluster size. Navigation is achieved via menus that order clusters by statistical significance (for BB clusters) or cluster size (for SC clusters, in which all members contain the sequence motif), and motif statistics are provided, along with text annotations for a selection of key motifs. In the figure, the D3S + 1 motif’s most significant BB cluster is selected along with its largest SC cluster, and the SC cluster viewer at right shows that these choices are associated with SC/SC and SC/BB H-bonds linking the turn to the bulge in the common type 1 beta-bulge loop (Milner-White 1987). The lower-level screen, opened by the button above the SC cluster viewer, supports the navigation of the “tail” clusters (contexts) associated with the sequence motif within the BB cluster selected on the upper-level screen, as well as the browsing and profiling of the underlying PDB structures.

The map interface, which is presented across two web pages, reflects the clustering hierarchy: the upper-level screen (Fig. 1) supports browsing the distributions of BB and SC structures associated with the motif, while the lower-level screen enables the exploration of the turn contexts, represented by the tail clusters, which are associated with the BB cluster selected on the upper-level screen. The lower-level screen also supports browsing and profiling the underlying PDB structures (Berman *et al.* 2000).

Clusters are displayed in JSmol viewers (Herráez 2006) on each map page, represented by the structures of their medoids, which are color-coded as a heatmap of motif statistical significance (at the BB level) or cluster size (at the SC and tail levels, where all cluster members contain the sequence motif), and scaled according to cluster size. At the SC level, the medoid structures convey the rotamer distributions of the motif’s SC(s). All structures are displayed in a turn-local coordinate system (Newell 2024b) which supports their visualization and comparison and implicitly aligns the global set of turns (Supplementary Section S1.2).

Maps also display the frequency distributions of the SC/SC, SC/BB, and BB/BB hydrogen bonds associated with the sequence motif, in the form of heat-mapped H-bonds connecting the corresponding atoms of the cluster medoids (see caveats in Supplementary Section S1.4).

Navigation in a map is achieved via menus listing the clusters at each level. Menu entries are ordered in the same way that medoids are heat-mapped—by the motifs’ statistical significance in each cluster (for BB clusters) or cluster size (for SC and tail clusters), enabling the rapid identification and browsing of important conformations at each level. Maps also incorporate embedded motif statistics, lists of the PDB addresses of the medoids and cluster members, lists of the PDB IDs alone for use in downloading structure files from www.rcsb.org/downloads, log-odds sequence profiles for the SC clusters within each BB cluster, and text annotations for a selection of notable sequence motifs.

Motif maps support the rapid and intuitive exploration of the recurrent structures and interactions associated with all single-AA and pair sequence motifs with sufficient abundance in beta turns and their BB contexts, along with many triplet motifs. Extensive built-in help is provided, including text prompts, a primer with annotated map pages, and an in-depth user guide. Documentation is also provided for the three beta-turn classification systems used in the maps, which partition the global set of turns at different levels of precision. In increasing order of precision, these systems include the nine classical types (Hutchinson and Thornton 1996) the 20 BB clusters (Shapovalov *et al.* 2019), and the Ramachandran-space-labeled types (Wilmot and Thornton 1990, Hollingsworth and Karplus 2010, Shapovalov *et al.* 2019), of which 351 occur in the dataset. The set of BB clusters is larger by two members than the set originally derived (Shapovalov *et al.* 2019), because the largest original cluster (“AD”), which represents classical type I and contains about half of all turns, is split into three parts to improve structural discrimination in the maps.

Motif-detection utilities available with MapTurns identify and rank the most statistically significant sequence motifs in each turn type, BB cluster or the global turn set. Motifs that are important in particular turn contexts, including helix caps (Aurora and Rose 1998, Newell 2015), beta hairpins (DuPai *et al.* 2021), supersecondary structures in which turns link helices/strands into particular geometries, or H-bonded BB motifs such as the Schellman loop (Schellman 1980) or the beta-bulge loops (Milner-White 1987, Newell 2024a) can be identified with the ExploreTurns tool (Newell 2024a), also available at www.betaturn.com. ExploreTurns also provides wild-card search features that rank the single-AA and pair sequence motifs present at particular positions or position ranges in turns/tails, as well as a feature that ranks the sequence motifs present in a turn/tail sequence entered by the user.

A broad, map-based survey of structure and interaction in beta turns and their BB neighborhoods is available with MapTurns, and serves as a “thumbnail index” to a selection of the most significant or otherwise notable SC motifs in turns and their contexts.

## 3 Implementation

The turn datasets, turn-local coordinate system, clustering methods, and the statistical techniques used to evaluate and rank sequence motifs (Hu *et al.* 1993, Bishop *et al.* 2007, Newell 2015) are described in the Supplementary File. Maps are implemented in HTML/CSS and Javascript, with embedded JSmol viewers. For best performance, browse with Chrome or Edge.

## 4 Applications

Since beta turns adopt a broad range of BB geometries and exhibit a wide variety of SC interactions, including H-bonds, salt bridges and hydrophobic, aromatic-Pro, pi-stacking, pi-(peptide bond) and cation-pi interactions (see the motif survey), they can serve as a laboratory for the general study of these interactions, and motif maps support this work. Maps characterize many new SC motifs and provide detailed structural rationalizations of sequence preferences, and the turn-local Euclidean-space coordinate system they incorporate enables the easy visualization and comparison of BB and SC conformations, which should make them a useful tool for education as well as research.

Maps also support mutational analysis, since a comparison of the maps for the motifs present in wild-type and mutant structures reveals the changes in structure and interaction that can occur with AA substitution(s). For example, a comparison of the map for the motif which specifies aspartic acid at the first turn position and arginine at the third position (D1R3, which forms one of the most significant salt bridges in beta turns), with the map for the motif which substitutes lysine at the third position (D1K3) shows the structural effect of the substitution: in BB cluster AA_1a(I), in which both motifs are most commonly found and short salt bridges are frequent in the two largest SC clusters in D1R3’s map, the lysine SCs in the largest SC clusters in D1K3’s map instead project away from D1, and the map shows no bridging in these clusters above the 20% display threshold. The map also shows the structural compromises that must be made for salt bridging with D1K3: bridging can occur in the smallest BB cluster, but the Lys SC must bend sharply (the bridge H-bond appears unphysical in the map due to the ambiguity in H-atom labeling at the end of the Lys SC). The disruption of salt bridging in D1K3 compared to D1R3 is likely responsible for the motif’s much lower fractional overrepresentation in the BB cluster (10% compared to 59%).

Maps should prove useful in any application that can benefit from a comprehensive and detailed picture of structure and interaction in beta turns and their contexts. One promising application is the design of protein loops, in which turns constitute close to two-thirds of residues (Shapovalov *et al.* 2019), and in particular the design of loops at binding and active sites, where turns play key functional roles.

## Supplementary Material

btae741_Supplementary_Data

## Data Availability

All data that support the findings of this study are available from the Protein Data Bank at www.rcsb.org.
