# Supplementary material for: MapTurns: mapping the structure, H-bonding, and contexts of beta turns in proteins
Source: Bioinformatics. 2024 Dec 16;41(1):btae741. doi: 10.1093/bioinformatics/btae741 (PMC11671037; doi:10.1093/bioinformatics/btae741)
Supplement: btae741_Supplementary_Data [file btae741_supplementary_data.docx]

**MapTurns**

**Supplementary information**

**S1 | Methods**

**S1.1 Turn datasets**

Beta turns are defined here as four-residue BB segments with a distance of no more than 7Å between the alpha carbons of their first and fourth residues and central residues with DSSP^1^ codes outside the set {H, G, I, E} that specifies helix or strand^2^. Turn/tail structures were extracted from their PDB^3^ files with the aid of a list of peptide chains with a maximum mutual identity of 25% obtained from PISCES^4^. Since sequence motif detection identified multiple motif "artifacts" generated by remaining local redundancy in the data, an additional step of turn-local redundancy screening was applied: turn/tail structures were clustered and filtered to reduce redundancy within five residues of turns to 40% or less; this threshold was chosen because it effectively reduced artifacts while preserving a dataset of sufficient size.

Turn structures were screened for quality by requiring that there be no missing residues within 5 residues of any turn and excluding structures with bond lengths that constituted outliers from established values. The final dataset of 102,192 turns was compiled with resolution and R-value cutoffs of 2.0Å and .25 respectively; average values in the dataset are 1.6Å and .20.

The three separate, smaller datasets of "strand" turns, for which one or more central residues are of DSSP type 'E', were generated with the same quality criteria as the beta-turn dataset. Maps for strand turns are labelled with the suffixes _E2, _E3, or _E2E3, indicating the residues that lie within strands; the corresponding turn datasets contain 5095, 3633 and 2982 structures respectively.

**S1.2 Turn-local coordinate system and alignment**

The common, turn-local Euclidean-space coordinate system which supports the second- and third-stage clustering used to generate motif maps, as well as the visualization and comparison of structures in the maps, was developed in a recent study by the present author, where it was applied to derive a set of geometric descriptors for beta turns^5,6^. The turn-local system is established via a set of geometric definitions: The **span line** is the line between a turn's first and last alpha carbons ($C_{\alpha1}$and $C_{\alpha4}$), and the span, already used in the beta-turn definition, is its length. The **turn center** is the midpoint of ($C_{2}$→$N_{3}$), the middle peptide bond in the turn. The **turn plane** contains the span line and passes through the turn center, and the perpendicular dropped from the turn center to the span line defines the **turn axis**. Using these definitions, a local, orthogonal right-handed coordinate system is established. The system's origin lies at the turn center, while the x axis lies in the turn plane, collinear with the turn axis, with its positive sense oriented from the center towards the span line. The y axis lies in the turn plane, parallel to the span, with its positive sense oriented towards the C-terminal half of the turn. Finally, the z axis is perpendicular to the turn plane, with its positive sense determined by the cross-product of the x and y axes.
 The atomic coordinates of each turn are transformed from the global protein frame in the turn's PDB file to the turn-local frame, implicitly aligning the structures. The alignment's accuracy was tested by a comparison between implicit pairwise turn alignments and the best alignments generated by an RMSD-based perturbative search procedure. In this process, the average distance between the corresponding BB atoms of randomly chosen, implicitly aligned turn pairs were compared to the distance obtained for the best alignments generated by a perturbative search applied to the pairs which seeks the minimum average interatomic distances by introducing small variations in the relative position and orientation of the two turns. This procedure was applied to a random sample of 100 million turn pairs to generate an estimate of the average positional error of the implicit alignments. Since this estimate (0.17 Å) is comparable to the errors expected for the positions of well-determined atoms in well-refined structures (0.1 Å - 0.2 Å; overall RMSDs for independently-determined 2.0 Å structures have been measured as 0.5 Å - 0.8 Å)^7^, the accuracy of the implicitly established alignment was judged to be satisfactory.

**S1.3 Clustering**

Motif maps are composed of medoids generated by three-stage hierarchical clustering. The first (BB) clustering stage employs a previously-derived set of medoids generated from an ultra-high-resolution dataset that represents all beta turns, using a hybrid DBSCAN/k-medoids procedure in Ramachandran space^2^. This medoid set was modified slightly to improve the precision of map coverage by splitting the largest cluster, which represents classical type I and type I-adjacent turn geometries, into three parts {AA_1a(I), AD_1b(I), AD_1c(I)}. In first-stage clustering, the medoid set is used to partition the 102,192 structures in the dataset compiled for the present study into BB clusters, by applying the same Ramachandran-space distance metric which was employed to generate the medoids^2^.

In second-stage, SC clustering, the structures in each BB cluster which contain the sequence motif (which have been aligned in Euclidean space by the implicit procedure described above) are clustered by applying the k-medoids PAM (Partitioning Around Medoids) algorithm^8^ in Euclidean space to the SC and BB atoms of the residues specified in the sequence motif, identifying the most important recurrent SC conformations.

Finally, third-stage (tail) clustering applies k-medoids PAM in Euclidean space to the BB atoms of the two-residue N- and C-tails of the turns in each SC cluster, identifying the most important recurrent contexts associated with the motif in each cluster. Clusters at all levels are represented in the JSmol viewers by their medoids, or, when tails are displayed, by composites of medoids; see the primer and user guide for details.

Four medoids are used in second-stage (SC) clustering to represent the SC structures in each BB cluster, while up to five medoids are used to represent the BB structures in the turn contexts (N- and C-tails) at the third clustering stage (given sufficient structure counts in the SC clusters). These medoid counts were determined by a trial-and-error procedure which sought to satisfy three goals: maps should distinguish between SC rotamers in second stage clustering, identify secondary structures in the turn's contexts (tails) in third-stage clustering, and provide a clear presentation of structure which avoids excessive detail which can obscure the most important structural distinctions. Silhouette analysis^9^ was not applied to select the number of clusters, due to the very large number of separate clusterings required to generate the maps (3200 for single-AA motifs in second-stage clustering alone, and a much larger number for the pair motifs), as well as the need to satisfy the goals described above, which require the application of domain knowledge regarding SC rotamers and secondary structures.

Clustering in the datasets of E2-, E3- and E2E3-strand turns applies the same methods as those used for beta turns, except that the first-stage (BB) clusters are generated by a k-medoids PAM algorithm in Ramachandran space, with the number of clusters determined by silhouette analysis combined with domain knowledge; first-stage medoids had to be generated for the strand turns because these turns were not included in the Ramachandran-space DBSCAN/k-medoids clustering carried out for beta turns^2^.

**S1.4 H-bond display**

The distribution of SC and BB H-bond frequencies within and between clusters is displayed in a map in the form of heat-mapped dashed lines between the corresponding donor and acceptor atoms in the cluster medoids; frequencies from 20-100% are displayed using the color scale shown in the map. The criteria used to define H-bonds are given in the user guide. The heat-mapped H-bonds may appear unphysical due to differences in conformation between a cluster's medoid and its members or ambiguities in atomic labelling, and atomic labelling ambiguities may also produce inaccuracies in the displayed H-bond frequency distributions.

The set of H-bonds displayed by JSmol in PDB structures does not always exactly correspond to the H-bonds shown in the maps, due to differences in the H-bond definitions applied by MapTurns and JSmol (for example, JSmol does not apply separate cutoffs for the H-bond angles centered on the H atom and the acceptor atom).

**S1.5 Sequence profiles**

Sequence profiles are provided for each SC cluster within each BB cluster in a map, in the statistics window on the lower-level map page. Profiles are log-odds scoring matrices^10^ which score the 20 AA types at each position in the turn/tails according to the frequency with which they occur in the cluster. The log-odds for AA type *i* at position *j* is:

$$L_{ij}=\log_{2} \frac{M_{ij}}{N_{i}}$$

where $M_{ij}$ is the abundance fraction of AA type *i* at position *j* in the cluster, and $N_{i}$ is the abundance fraction of type *i* at any position in the complete set of protein chains used to construct the MapTurns/ExploreTurns database.

**S1.6 Computing sequence motif overrepresentation and p-value**

Statistical tools^5,6,11,12,13^ are used in motif maps to evaluate the over/under-representation and the significance of sequence motifs in turn types and BB clusters, and the chi-square measure of significance is heat-mapped onto the BB medoids in the BB cluster view. The degree of over/under-representation of a motif is measured by its fractional overrepresentation $\left( O-E \right)/E$, where O is the motif's observed count in the set and $E$ is its expected count under a suitable null model. The statistical significance of a motif in a selected set of structures is measured by its p-value, which represents the probability that the motif's departure from its expected count would be as large or larger than its actual value by chance alone under the null model. Lower p-values indicate a smaller chance that the null model is consistent with the data, and consequently a greater chance that the model is incorrect and the motif is significantly over- or underrepresented.

For a single-AA sequence motif, the null model specifies that the probability $P$ of the motif's occurrence in the set is equal to the position-independent probability of the occurrence of the motif's AA anywhere in proteins, which is computed as the overall abundance fraction of the AA in the complete dataset of protein chains used in the study: $P= {C_{AA}}/T$, where $C_{AA}$ is the count of the AA in the set of all chains and $T$ is the total number of residues in the chain set. The motif's expected count in the set is then $E=NP$, where $N$ is the size of the set. The p-value is computed as the combined area underneath the two tails of the binomial distribution with parameters $N$ and $P$ that represents the null model of random motif occurrence; the tails represent the probability that the motif's observed count would equal or exceed its value by chance alone under the model.

A sequence motif which specifies two AAs at two positions (pair motif) is evaluated by measuring the significance of the two-factor effect in a 2x2 contingency table in which the rows of the table indicate the presence or absence of the AA specified at the motif's first position and the columns indicate the presence/absence at its second position. For example, the following contingency table might be used to evaluate the pair motif D1P2 that specifies Asp at turn position 1 and Pro at turn position 2 in a set of 100 turns:

|  | $Pro2$ | $\bar{Pro2}$ |
| --- | --- | --- |
| $Asp1$ | 10 | 15 |
| $\bar{Asp1}$ | 20 | 55 |

Here the labels indicate the presence or absence (barred label) of each AA at its position, and the table contains the observed counts $O_{ij}$for each of the four possible combinations of presence/absence of the two AAs at positions 1 and 2; when D1P2 is present in a turn, it is counted in cell (1,1) of this table.

The null model for a pair motif specifies independent occurrence of each AA at each position, which indicates no pair synergy between the motif's individual components. Under this model, the probability that a turn falls into cell ($i,j$) of the 2x2 observed table, where $i$ indexes the rows and $j$ the columns, is:

$$\begin{aligned} P_{ij}=P_{1\left( i \right)}\times P_{2\left( j \right)}=\frac{M_{1\left( i \right)}}{N}\times\frac{M_{2\left( j \right)}}{N} \end{aligned}$$

where $P_{1(i)}$ represents the probabilities of the turn either containing the first AA specified by the motif at its particular turn position ($i = 1$) or not containing that AA at that position ($i = 2$), and $P_{2(j)}$ represents the independent probability of the presence or absence of the second AA at its position. $M_{1(i)}$ and $M_{2(j)}$ are the table's margin totals for its rows ($M_{1(i)}$), formed by summing the table over its columns, and its columns ($M_{2(j)}$), formed by summation over its rows, and $N$ represents the total number of structures in the set.

The expected counts for the cells of the 2x2 table under the null hypothesis of independence are:

$$\begin{aligned} E_{ij}={N\times P}_{ij}=\frac{M_{1\left( i \right)}\times M_{2\left( j \right)}}{N} \end{aligned}$$

so the expected count for the motif, in cell (1,1) of its table, is:

$$\begin{aligned} E_{11}=\frac{M_{1\left( 1 \right)}\times M_{2\left( 1 \right)}}{N} \end{aligned}$$

and its fractional overrepresentation is:

$$\begin{aligned} \frac{\left( O_{11}-E_{11} \right)}{E_{11}} \end{aligned}$$

where $O_{11}$ is the motif's observed count.

To compute the motif's p-value, a chi-squared metric is computed which measures the distance between the observed counts in the four cells of the motif's 2x2 table and their corresponding expected counts:

$$\begin{aligned} \chi^{2}=\sum_{i=1}^{2} \sum_{j=1}^{2} \frac{\left( O_{ij}-E_{ij} \right)^{2}}{E_{ij}} \end{aligned}$$

The p-value is obtained by comparison of this metric with the chi-squared distribution with a single degree of freedom which represents the two-factor effect, or pair synergy, present in the observed table. P-values are not corrected for multiple testing error so that they may be used as reference values for individual particular motifs of interest.

Triplet motifs are evaluated using a method analogous to that applied for pair motifs. For a triplet motif in a turn set, the null model specifies no three-factor effect in the 2x2x2 contingency table for the motif, in which each dimension represents the presence or absence of one of the three individual AA components in the motif. This model specifies that there is no triplet synergy associated with the motif; that is, there is no association between any pair of AAs in a motif and the remaining, single AA.

The expected counts for the null model of no three-factor effect in a triplet motif's 2x2x2 observed table cannot be computed using a closed-form expression such as that used to calculate $E_{ij}$ in the 2x2 tables of pair motifs, but instead must be calculated via iterative proportional fitting (IPF)^11^, which begins with a table of uniform estimated expected counts and applies, at each iteration, each of the three 2x2 tables of margin totals computed from the motif's 2x2x2 observed table to this table of estimates, modifying the estimates in each step until they converge with a desired level of precision. The resulting expected count estimates are consistent with all three observed 2x2 margin tables, so they contain all three two-factor effects in the data (along with the three single-factor effects associated with the individual AAs), and they represent the maximum likelihood estimates for the expected counts, under the null model which lacks only the three-factor effect.

When a triplet motif is present in a turn, it is tabulated in cell (1, 1, 1) of its observed 2x2x2 table, so the motif's expected count is $E_{111}$, and its fractional overrepresentation is:

$$\begin{aligned} \frac{\left( O_{111}-E_{111} \right)}{E_{111}} \end{aligned}$$

where $O_{111}$is the motif's observed count.

To compute the motif's p-value, a chi-squared metric is computed to measure the distance between the observed counts in the eight cells of the motif's 2x2x2 observed table and their corresponding expected counts:

$$\begin{aligned} \chi^{2}=\sum_{i=1}^{2} \sum_{j=1}^{2} \sum_{k=1}^{2} \frac{\left( O_{ijk}-E_{ijk} \right)^{2}}{E_{ijk}} \end{aligned}$$

The motif's p-value is obtained by comparison of this metric with the chi-squared distribution with one degree of freedom, which represents the (single) three-factor effect, or triplet synergy present in the observed table. P-values are not corrected for multiple testing error so that they may be used as reference values for individual motifs of interest.

Note that the absence of synergy in a pair motif means only that each AA in the motif occurs independently of the other, and that the motif is therefore not likely to represent a significant interaction between the two AAs. This means that the effect of the simultaneous occurrence of the two AAs is likely to be no more important than the sum of their individual effects, but it does not mean that the occurrence of the pair has no importance, since the pair is an instance of both single-AA motifs, each of which may themselves be significant. In order to fully evaluate the importance of a pair motif, it is therefore necessary to consider the importance of its components; for example, the D1R3 motif is overrepresented in type I turns by 45%, likely reflecting its associated intra-turn salt bridges, but its D1 component is overrepresented in the type by 159%, reflecting the SC/BB H-bonding that makes D1 the most important of all H-bond motifs in the type. The analogous consideration applies to triplet motifs: the absence of triplet synergy means only that the motif is not likely to represent a significant interaction between its pair components and the single-AA components which complement them; it does not necessarily mean that the occurrence of the triplet has no importance, since it is also an instance of its underlying pair and single-AA motifs.

**References**

1) Kabsch W, Sander C. Dictionary of protein secondary structure: pattern recognition of hydrogen-bonded and geometrical features. *Biopolymers*. 1983;22(12):2577-637.

2) Shapovalov M, Vucetic S, Dunbrack RL. A new clustering and nomenclature for beta turns derived from high-resolution protein structures. *PLOS Computational Biology*. 2019;15(3).

3) Berman HM, WestBrook J, Feng Z, Gilliland G, Bhat TN et al. The Protein Data Bank. *Nucleic Acids Res*. 2000;28(1):235-242.

4) Wang G, Dunbrack RL. PISCES: A protein sequence culling server. *Bioinformatics*. 2003;19(12):1589-91.

5) Newell NE. Geometric descriptors for beta turns. *Protein Science*. 2024;33(9):e5159.

6) Newell NE. A geometric parameterization for beta turns. *bioRxiv*. doi: https://doi.org/10.1101/2024.01.01.573818

7) Richardson JS. The anatomy and taxonomy of Protein structure. *Adv. Protein Chem*. 1981; 34:167-339.

8) Kaufman L, Rousseeuw, PJ. Partitioning Around Medoids (Program PAM). In: Kaufman L, Rousseeuw PJ, *Finding Groups in Data: An Introduction to Cluster Analysis*. Hoboken: Wiley-Interscience, 2005, 68-125.

9) Rousseeuw, PJ. Silhouettes: A graphical aid to the interpretation and validation of cluster analysis. *Journal of Computational and Applied Mathematics*. 1987; 20:53-65.

10) Ogilvie, H. Position-specific score matrices. https://cs.rice.edu/~ogilvie/comp571/pssm/, retrieved Oct. 12, 2024.

11) Bishop YM, Fienberg SE, Holland PW. Discrete Multivariate Analysis: Theory and Practice. New York: Springer; 2007.

12) Hu JC, Newell NE, Tidor B, Sauer RT. Probing the roles of residues at the e and g positions of the GCN4 leucine zipper by combinatorial mutagenesis. *Protein Science*. 1993;2(7):1072-84.

13) Newell NE. Mapping side chain interactions at protein helix termini. *BMC Bioinformatics*. 2015;16:231.
